# Role of the Heart in Lactate Shuttling

**DOI:** 10.3389/fnut.2021.663560

**Published:** 2021-04-22

**Authors:** George A. Brooks

**Affiliations:** Exercise Physiology Laboratory, Department of Integrative Biology, University of California, Berkeley, Berkeley, CA, United States

**Keywords:** heart, lactate, glucose, fatty acids, ketones, exercise, metabolism, muscle

## Abstract

After almost a century of misunderstanding, it is time to appreciate that lactate shuttling is an important feature of energy flux and metabolic regulation that involves a complex series of metabolic, neuroendocrine, cardiovascular, and cardiac events *in vivo*. Cell–cell and intracellular lactate shuttles in the heart and between the heart and other tissues fulfill essential purposes of energy substrate production and distribution as well as cell signaling under fully aerobic conditions. Recognition of lactate shuttling came first in studies of physical exercise where the roles of driver (producer) and recipient (consumer) cells and tissues were obvious. One powerful example of cell–cell lactate shuttling was the exchange of carbohydrate energy in the form of lactate between working limb skeletal muscle and the heart. The exchange of mass represented a conservation of mass that required the integration of neuroendocrine, autoregulatory, and cardiovascular systems. Now, with greater scrutiny and recognition of the effect of the cardiac cycle on myocardial blood flow, there brings an appreciation that metabolic fluxes must accommodate to pressure-flow realities within an organ in which they occur. Therefore, the presence of an intra-cardiac lactate shuttle is posited to explain how cardiac mechanics and metabolism are synchronized. Specifically, interruption of blood flow during the isotonic phase of systole is supported by glycolysis and subsequent return of blood flow during diastole allows for recovery sustained by oxidative metabolism.

## Introduction

The purported role of lactate in physiology and medicine is a century old, but understanding of the role has changed dramatically in the last three decades (1–7). No longer thought of as a dead-end metabolite, a fatigue agent, and metabolic poison, in contemporary physiology, lactate is seen as a major metabolic intermediate that has wide-ranging impacts in energy substrate distribution and utilization, gluconeogenesis, and cell signaling (1, 2, 5, 6). While extensive data have been presented to support the lactate shuttle hypothesis in humans *in vivo*, little had been written to describe the role of lactate in cardiac metabolism or the role of the heart in terms of overall, whole body–energy substrate balance. Beyond the important role of lactate in supporting cardiac functioning, examination of the role of lactate in cardiac metabolism is illustrative overall regulation of energy substrate partitioning in other body tissues and organs.

In terms of fuel energy substrate use, the heart is sometimes referred to as “omnivorous,” meaning it can simultaneously oxidize a variety of energy substrates including glucose, lactate, fatty acids, and ketones. The heart is also regarded as a “pay as you go” energy consumer because it relies heavily on exogenous as opposed to endogenous energy sources. As previously shown for skeletal muscle and whole-body metabolism (8), changes in cardiac work determine the rates and relative uses of energy substrates, with lesser work emphasizing lipid oxidation (9–12) and greater work emphasizing carbohydrate (i.e., glucose and lactate) energy sources (10, 12). The cardiac glycogen pool probably turns over, just as in skeletal muscle (13), but degradation in excess of synthesis is not as exaggerated as in working skeletal muscle (14). Hence, glycogen is not known to be a major energy source for the healthy heart. And strictly speaking in terms of the mobilization of endogenous energy sources, it is apparent that mobilization of intramuscular triglyceride can result in mobilization of fatty acids within the heart even though it is a net consumer of fatty acids from the systemic circulation (11).

A unique reason to emphasize cardiac lactate metabolism in this paper is that of the purported functions of lactate shuttling (energy substrate, gluconeogenic precursor, signaling molecule), studies indicate that the heart depends heavily on exogenous lactate as a fuel when cardiac work is elevated. In contrast to the liver and kidneys, the heart is not a gluconeogenic organ. And while the heart continuously receives neuro-endocrine signaling from the vagus and cardiac nerves as well as arterial blood, the heart also releases atrial natriuretic peptide (ANP), a unique endocrine signaling function of the heart induced by mechanical atrial stretching when blood volume is elevated. ANP secretion is not known to involve lactate signaling.

### Lactate Shuttling: Roles of Driver and Recipient Cells

Before getting into specifics, it is advisable to introduce general concepts of lactate shuttling. Key to shuttling is that there exist lactate producing (driver) and recipient (consuming) cells and tissues (1, 6, 15). Experimentally, the shuttle concept was supported by findings that lactate flux between cells and tissue beds depends on metabolite and hydrogen ion (pH) concentration differences (16–19). Lactate exchanges between and among cells were shown to be facilitated by the presence of cell membrane lactate transport proteins termed monocarboxylate transporters (MCTs) (19, 20); MCTs are bidirectional symporters (16, 17, 21) sensitive to trans-stimulation by lactate and hydrogen ion gradients. Importantly, based on the results of studies on rat muscles after exercise (22–24), dog gracilis muscle preparations (25), and subsequently studies on resting and exercising human skeletal muscles (26, 27) and hearts (28, 29), the lactate shuttle concept emerged (1, 6, 15). Not surprisingly, we now know that driver and recipient cells can switch roles depending on conditions, and that some cells can exchange lactate through the interstitium and vascular beds. For instance, during exercise, fast white fibers can provide oxidizable substrate to red, oxidative fibers in the same tissue bed (22, 30). Conversely, postprandial glucose uptake in red fibers can provide substrate to the body corpus as in the glucose paradox (31) and postprandial lactate shuttle (32). By means of lactate shuttling, working muscle can fuel the beating heart (28), support cerebral executive functions (33–39), and provide gluconeogenic precursor to the splanchnic organs (40, 41).

### The Heart Is Difficult to Access for Study

**Figure 1:**
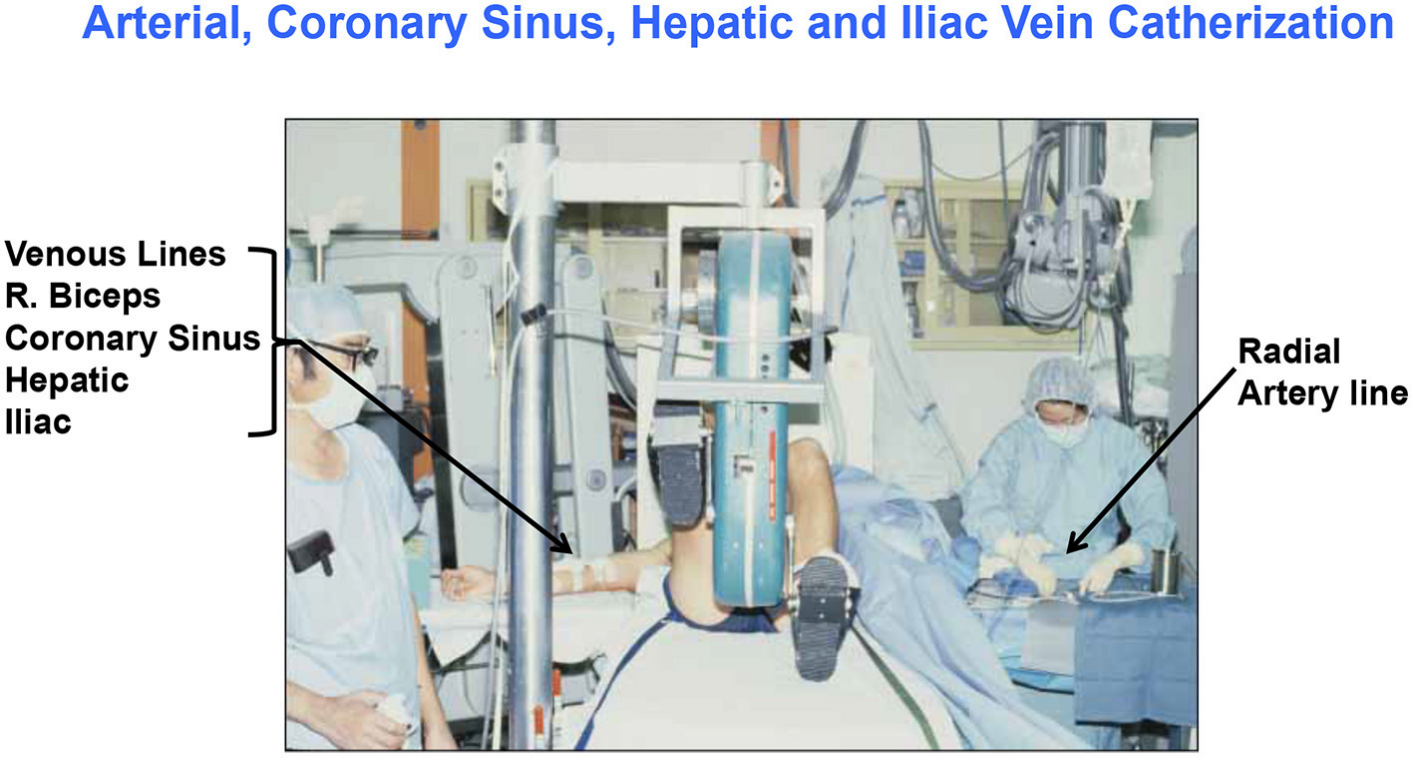
Illustration of the invasive and complicated methods to simultaneously determine cardiac and whole-body energy substrate partitioning in man. Setting for (26, 28, 29, 45–47); G. A. B. personal photo.

Historically, investigators have been challenged in studying cardiac metabolism because substrate balance studies require making blood flow and arterial-venous difference (a-v) measurements. Arterial catheterization is considered to be invasive, but perhaps more invasive still is sampling venous output from the heart, which involves catheterization of the coronary sinus (CS), a vessel that empties into the right atrium. Hence, much of the data were obtained on patients with heart or other diseases receiving appropriate medications as contrasted to healthy young individuals (10, 42–44). Fortunately, there are studies on healthy humans (typically men) involving CS catheterization with blood flow and metabolite concentration measurements (28, 29, 45). Fortunately, there are also studies on healthy men and women that applied strict dietary controls required for interpretation of metabolism data (11, 12). An example of an experimental setting for cardiac and whole-body measurements of energy–substrate partitioning in a resting and exercising human subject is shown in Figure 1.

In addition to problems related to arterial and CS access and metabolite as well as blood flow measurements, other problems are related to the assessment of metabolites that undergo turnover within the tissue or organ of interest. Because glucose is taken up by, but not produced in, the myocardium, simple net chemical balance measurements, i.e., [(a-v) Metabolite (blood flow)], are adequate to assess cardiac glucose metabolism. However, such measurements are not adequate for lactate because it is simultaneously taken up, produced, and oxidized within the heart (12, 28). Also, the same problem applies to determinations of fatty acid metabolism, although to a lesser extent (11).

**Figure 2:**
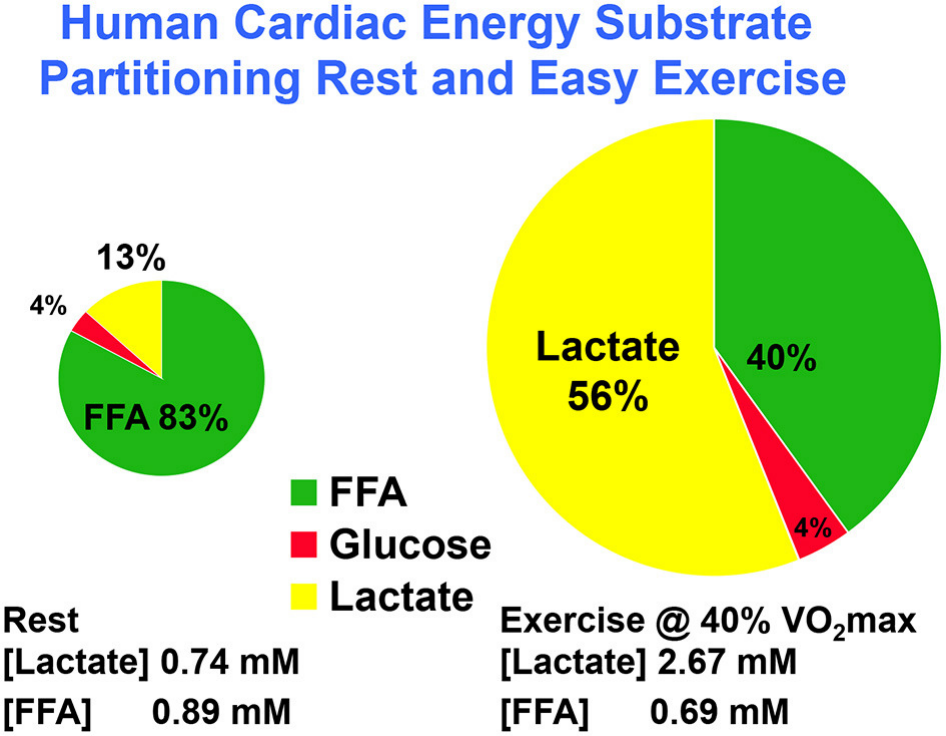
Illustration of substrate–substrate interactions in cardiac metabolism as determined in a man resting or engaged in easy, 40% VO_2_max exercise. Results show that the contribution of lactate to cardiac energy need depends on arterial lactate concentration and is significantly greater than that of glucose. Results also show crossover from fatty acid to lactate dependence in heart during exercise. Data from (28).

Using coronary sinus catheterization methodology, early investigators noted the omnivorous capabilities of human hearts, as well as the ability of one substrate, such as glucose, to suppress use of another, such as fatty acids (10, 48). Thus, early investigators making measurements of arterial-coronary sinus concentration differences, coronary flow, and oxygen extraction could determine energy substrate partitioning in human hearts. Subsequent experiments with isotopic tracer verified preferential myocardial FFA utilization (11, 45) and that FFA availability downregulates glucose utilization, and *vice versa* (45, 49). For the later experiments, investigators had the advantage of using radioactive and stable lactate isotope tracers that revealed significant intra-organ turnover. Moreover, studies using physical exercise (28, 29, 45) or cardiac pacing (11, 12) showed crossover from lipid to carbohydrate energy sources with increasing cardiac work. Thus, with measurements of cardiac metabolism in the setting of leg cycle ergometer exercise, it could be observed that lactate released from muscle and other driver sites became the major cardiac energy source in healthy young men as illustrated in Figure 2, an example of cell–cell lactate shuttling.

### Lactate and Ketones, Alternative or Major Energy Substrates?

With few exceptions, extant data on resting humans give rise to the conclusion that fatty acids predominate over glucose as the major fuel energy course for cardiac metabolism in resting, healthy individuals. Hence, fatty acids and glucose are typically considered to be major fuel energy substrates for the heart. For instance, in overnight fasted healthy individuals following an American Heart Association (AHA) compliant diet, the cardiac respiratory quotient (RQ) and whole-body respiratory exchange ratio (RER = VCO_2_/VO_2_) averaged 0.83, or 58% lipid and 42% carbohydrate energy utilization. In this setting, beta-hydroxybutyrate and acetoacetate (i.e., “ketone bodies,” derivatives of lipid catabolism), and lactate (a derivative of glucose metabolism) are sometimes considered to be “alternative” energy substrates.

Initial studies of cardiac metabolism demonstrating utilization of FFA and ketones over glucose were conducted under carefully controlled conditions prudent considering invasiveness of the procedures and medical conditions of subjects (10, 48). However, supine rest is not typical of many activities of daily life such as requiring increased cardiac work. As illustrated in Figure 2, the notion of lactate as an alternative cardiac fuel energy source comes to question. During whole-body exercise, lactate is not an alternative fuel energy source, *lactate is the main cardiac energy fuel the utilization of which downregulates fatty acid uptake and metabolism* (28, 29, 45, 50). Interestingly, the same dominance of lactate over glucose and fatty acids as brain energy sources has been demonstrated in traumatic brain injury (TBI) patients and healthy controls (35, 51) as well as at the whole-body level during physical exercise (27, 52, 53).

Given the absolute and relative increase in cardiac lactate metabolism in the transition from rest to moderate-intensity supine physical exercise (Figure 2) (29, 54) or electrical pacing to heart rate 120 bpm (12), one wonders what energy substrate partitioning would be during hard, upright physical exercise for instance at 65–70% VO_2_max. At present, such data are unavailable, but given the known relationships among exercise intensity, circulating lactate level, and lactate uptake and oxidation determined across working muscle beds (27) and at the whole-body level (27, 46, 55, 56), a reasonable short-term prediction is that during hard exercise when arterial blood lactate concentration rises, myocardial lactate uptake and oxidation would rise also.

Beyond a predicted acute, short-term prediction of an effect of lactate myocardial energy substrate partitioning is an observed effect of lactate on MLOC constituent gene expression, in particular MCT1 and MCT4, as well as LDH that were observed in response to lactate incubation in perfused rat myocardium (57). As noted previously with L6 myocytes, lactate incubation appeared to work via PGC1α, ROS, and NRF mechanisms (58). Should repeated lactate exposures as would occur with regular exercise training, a long-term prediction would be an enhancement of myocardial capacity for lactate uptake, facilitated by increased MCT1 and MCT4 protein abundances, and lactate oxidation as facilitated by increased MMCT, LDH, and other MLOC protein levels.

Still, even in the absence of data obtained during hard, upright exercise, lactate may be considered to be the major fuel energy source for essential tissues such as brain, red skeletal muscle, and heart on conditions of increased energy demand.

### Glycolysis and Glycogenolysis in the Heart

Tracer studies and non-tracer (a-v) studies show that the healthy heart takes up lactate on a net basis because the highly expressed mitochondrial reticulum acts as a sink for substrate oxidation (12, 29). Also, studies on healthy humans show that the heart simultaneously extracts, produces, and oxidizes lactate especially when cardiac work is elevated (12, 28, 29). But, how can these apparently discordant results of simultaneous production and disposal be explained? This important question can be addressed in part by confirmative studies on heart and skeletal muscle preparations using ^13^C-lactate and nuclear magnetic resonance spectroscopy and hyperpolarized MRS (59–61). Given these observations, what are the mechanisms of glucose uptake, lactate formation, and lactate disposal?

The heart takes up and oxidizes glucose on a net basis because there is a need for energy substrate to support cardiac work, but also there appears to be a compartmental need, probably for excitation–contraction coupling (62). In the heart, glucose gives rise to lactate because it is the inevitable consequence of glycolysis (63), the minimal muscle L/P being 10 and rising to an order of magnitude or more when glycolytic flux is high (64). The heart produces lactate from glucose because the enzymes of glycolysis, including lactate dehydrogenase, are highly expressed in the myocardium and are up regulated in ischemic disease (65) and are associated with increased reliance of lactate as an energy source (29).

That the heart takes up glucose and produces lactate may be in part due to cardiac anatomy and limits to intra-cardiac circulation imposed by the cardiac cycle. If it is true that even in a healthy heart systole interrupts coronary blood flow, especially in the endocardium, then the presence of a phasic intra-cardiac lactate shuttle is indicated. Assuming a resting heart rate of 60Bpm, there occurs 200ms of stopped flow during the isometric contraction phase of systole which is powered by glycolysis followed by 800ms of diastole for oxidative recovery and lactate clearance. Admittedly, the presence of an intra-cardiac lactate shuttle would be difficult to prove because even coronary sinus sampling would be insufficient to determine phasic events. Hopefully in the future, gated hyperpolarized MRS (60) or other technologies will allow for detection of lactate production, net uptake, and oxidative disposal within a cardiac cycle.

Even after endurance training, working muscle relies on glycolysis from glucose, and more importantly, from glycogen (27, 66, 67). In contrast, in the absence of ischemia or systemic anoxia or exsanguination, cardiac muscle glycogen does not appear to change. However, it may well be that glycogen synthesis is continuous (13) and that cardiac glycogen turnover involves a glycogen shunt as described by Robert Shulman and colleagues (68).

### Cardiac Stress and Energy Substrate Crossover

As illustrated for physical exercise (28, 29) or cardiac pacing (11, 12), increased cardiac work shifts substrate selection from lipid- to carbohydrate-derived energy sources. Also, this switch or crossover from lipid to carbohydrate-derived energy sources is seen in other stressful conditions such as heart failure (69), diabetes (70), and aging (71). Teleologically, the crossover can be attributed to increased oxygen efficiency of carbohydrate-derived fuel energy sources (12, 72).

### Crossover Mechanisms, Lipid to Carbohydrate and Back

Mechanisms at both ends of the pathways involved in lipid metabolism, from lipolysis in white adipose tissue to mitochondrial fatty acid uptake and oxidation, explain crossover from lipid- to carbohydrate-derived energy sources in the stressed heart.

#### Substrate Availability, Lactate, and Lipolysis in Adipose

Inverse relationships between blood (La^−^) and plasma free fatty acid concentration (FFA) and oxidation during hard exercise has long been recognized (8, 73–75), an effect attributed to hydrogen ions or lactate anions that overcome the stimulatory effect of epinephrine on adipose lipolysis.

**Figure 3:**
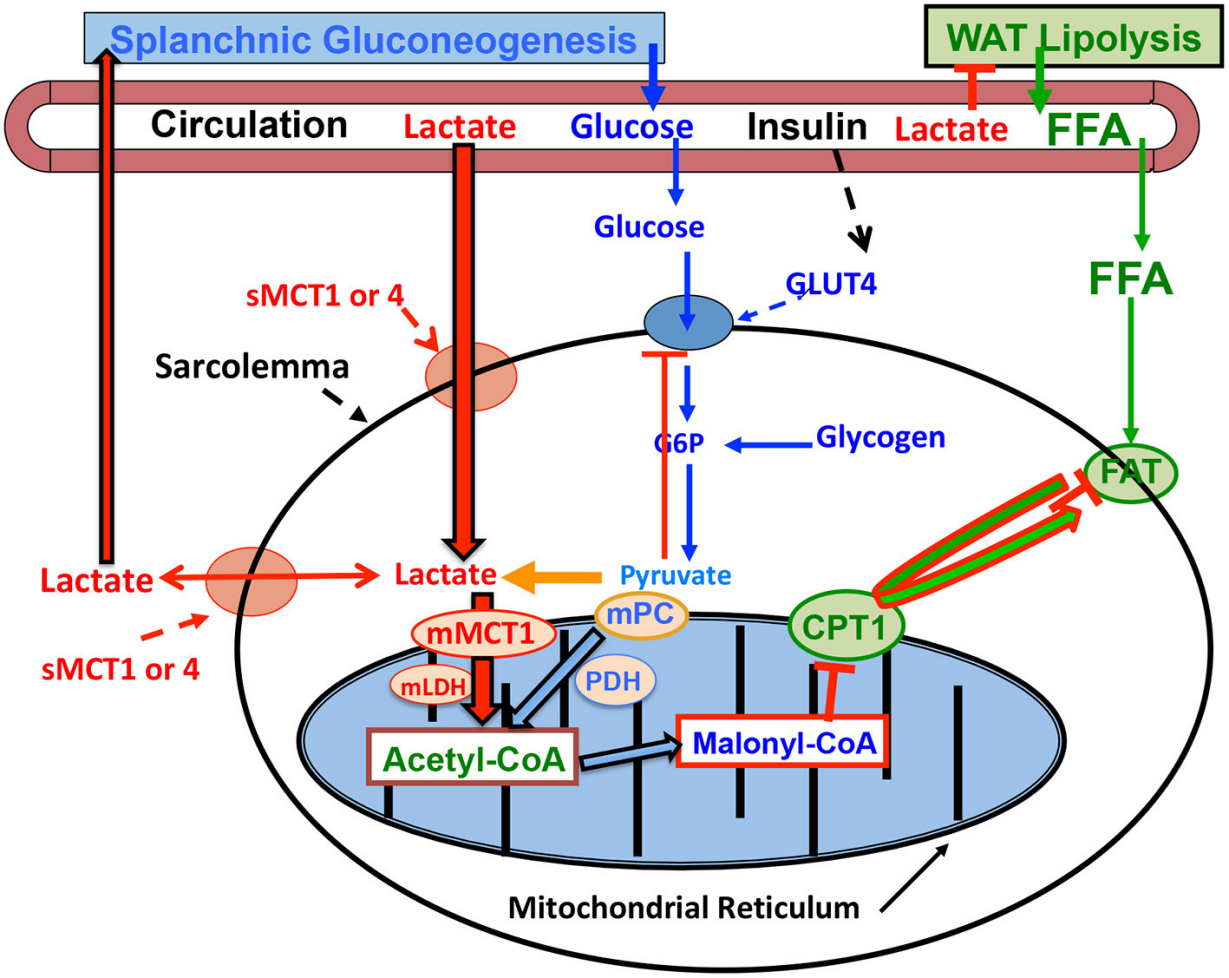
Illustration of how lactatemia affects crossover from lipid- to lactate-based metabolism in cardiac and skeletal muscle. Lactate is the inevitable consequence of glycolysis (63), the minimal muscle L/P being 10 and rising to an L/P >100 when glycolytic flux is high (64). Lactate availability as a myocardial energy source increases during physical exercise because arterial lactate concentration rises (27, 55), but also as the result of increased cardiac work and glycolytic flux during exercise (28, 29) or as the result of pacing (12). As the favored Oxidizable substrate, lactate catabolism results in product inhibition of glucose and FFA oxidation. As the products of glycolysis, lactate and pyruvate provide negative feedback inhibition of glucose disposal (blue dashed lines). Also as the predominant mitochondrial substrate, lactate gives rise to Acetyl-CoA, and in turn Malonyl-CoA. Acetyl-CoA inhibits β-ketothiolase, and hence β-oxidation, while Malonyl-CoA inhibits mitochondrial FFA-derivative uptake via CPT1 (**T**) (76). Moreover, lactate is the main gluconeogenic precursor raising glucose production and blood (glucose) (red lines). Via GPR81 binding, lactate inhibits lipolysis in WAT (**T**) depressing circulating (FFA) (77, 78). This model explains the paradoxical presence of lactatemia in high-intensity exercise and insulin-resistant states with limited ability to oxidize fat (green lines). Modified from Hashimoto et al. (79). CPT1, carnitine palmitoyl transporter-1; FFA, free fatty acid; FAT, fatty acid translocator composed of CD36 and FABPc; GLUT, glucose transporter; s, sarcolemmal; m, mitochondrial. Malonyl-CoA formed from exported TCA citrate controlled by the interactions of Malonyl-CoA decarboxylase (MCD) and acetyl-CoA carboxylase (ACC). MCT, monocarboxylate transporter; MPC, mitochondrial pyruvate transporter; PDH, pyruvate dehydrogenase; WAT, white adipose tissue; **T**, inhibition. Not shown is fatty acyl-Co (FA-CoA) that will accumulate if FFAs are taken up by myocytes, but blocked from mitochondrial entry by the effect of Malonyl-CoA on CPT1. Accumulated intracellular FA-CoA will give rise to intramyocellular triglyceride (IMTG) and the formulation of LC-FA, DAG, and ceramides via inhibition of PI3 kinase (PI3-k) and reducing GLUT4 translocation; from (1).

The mechanism by which lactatemia suppresses circulating FFA is diagrammed in Figure 3. It is now known that there is suppression of adipose lipolysis by lactate working through receptor binding (77, 80–82). Moreover, it is now known that, independent of pH, lactate inhibits lipolysis in fat cells through activation of a previously orphan G-protein coupled receptor (GPR81), now termed hydroxycarboxylic acid receptor 1 (HCAR-1). The effect of lactate binding to HCAR-1 operates through cyclic-AMP (cAMP) and CREB (78, 83, 84). Ultimately, lack of substrate availability limits use of blood-borne fatty acids when the heart is stressed in the presence of lactatemia, with lactate being an oxygen-efficient fuel energy source and an inhibitor of fatty acid availability (85) as annotated in Figure 2.

#### Lactate and Pyruvate as Inhibitors of Mitochondrial Oxidation

The crossover from lipid- to lactate-based myocardial lactate fueling during exercise illustrated in Figure 2 is explained as follows. When glycolysis is accelerated during skeletal muscle contraction, arterial lactate (L) and pyruvate (P) concentrations rise because of net release and raise the L/P an order of magnitude from a nominal value of 10 (86). Also, because of increased cardiac work and glycolytic flux during whole-body physical exercise (28, 29), or as the result of pacing (12), lactate availability to the cardiac muscle mitochondrial reticulum rises. Then, because of the relatively greater abundance of lactate compared to pyruvate, lactate becomes the main precursor of acetyl-Co-A formation (76, 87, 88) and, thereby, malonyl-CoA (Figure 3). The rise in malonyl-CoA inhibits the entry of activated FFAs into the mitochondrial matrix by inhibiting carnitine-palmitoyl transferase-1 (CPT1) (76, 89). Also, the accumulation of acetyl-CoA downregulates β-ketothiolase, the terminal and rate-limiting enzyme of the mitochondrial β-oxidation pathway. Moreover, increased cardiac frequency and work stimulates glycolysis, and by changing the L/P, inversely the NAD^+^/NADH, and consequently cytosolic redox. Hence, by mass action, allosteric binding, and effects on cell redox, lactate acts to shut the gates of activated fatty acids into matrix of the mitochondrial reticulum. Suppression of cardiac capacity of lipid oxidation may also be attributable to downregulation of enzymes of fatty acid oxidation (90).

### Role of the Heart in Whole-Body Lactate Shuttling

Extant data show simultaneous lactate uptake, production, and disposal in the healthy human heart. A question then is how much of whole-body lactate turnover is attributable to heart metabolism? For this, we have one complete study using electrically stimulated atrial cardiac pacing (i.e., pacing) as well as the ability to piece together results from a limited number of disparate physical exercise studies.

#### Atrial Pacing

For cardiac pacing, Bergman and colleagues studied subjects with primed continuous infusions of [3, 3, 3-^2^H] lactate and [6, 6-^2^H] glucose (12). Subjects were healthy, of moderate fitness (VO_2_max = 35.5 ± 3 ml/kg/min), were on an AHA-compliant diet, fed supper, and slept in the university General Clinical Research Center and studied in the morning after an overnight fast. Arterial and coronary sinus blood sampling and measurements of CS blood flow were made and allowed for simultaneous calculation of whole-body and cardiac metabolite flux rates. Compared with resting (≈70 beats min/min), heart rate increased (≈111 beats min/min) due to pacing. From rest, myocardial blood flow (≈200 ml/min) doubled while myocardial oxygen consumption at rest (22 ml/min) increased three times due to atrial pacing. Resting mean blood pressure (MAP≈100 mmHg) and cardiac output (5.3 L/min) did not change significantly from rest during pacing. Arterial levels of catecholamines, insulin, and glucagon were unchanged due to cardiac pacing. Stroke volume during rest (77 ml) declined 31% because cardiac output was unaffected by pacing.

#### Glucose

There was no arterial-coronary sinus difference (a-CS) for glucose isotopic enrichment across the heart during rest or pacing verifying the absence of glucose production in the organ. Thus, the product of (a-CS) for glucose and blood flow [(a-CS) Glucose (Blood Flow)] gave net glucose uptake. Whole-body and cardiac RQs (≈0.83) were predictable considering the controlled diet and fast, indicating a 58–42% lipid–CHO fuel mix that did not change due to pacing. Glucose fractional extraction during rest (9%) was maintained during pacing, but blood flow doubled indicating that net cardiac glucose uptake (≈14 mg/min) increased 60% due to pacing (12).

#### Lactate

The arterial-coronary sinus difference (a-CS, 0.35mMol/L) was maintained in the transition from rest to pacing, indicating myocardial net lactate uptake under both resting and paced conditions. Isotopically measured fractional lactate extraction (≈65%) was maintained during pacing, but isotopic enrichment in CS blood declined 50% indicating major intra-organ lactate production. Consequently, myocardial lactate production during pacing increased 230% (i.e., was 3.3-fold over rest values). Thus, the heart accounted for a significantly greater percentage of whole-body lactate Disposal during atrial pacing (15%) compared with rest (5%) (12). Those percentages were similar to those found previously (29, 47). In Bergman et al., investigators used deuterated as opposed to ^13^C- or ^14^C-lactate tracer previously (27). Consequently, lactate oxidation rate could not be determined. However, in previous studies using carbon-labeled tracers on the heart (29) or skeletal muscle (27), most lactate taken up was oxidized *in situ*.

#### Whole-Body Exercise

Atrial pacing studies serve to illustrate dynamics under tightly controlled conditions. However, during physical exercise, far greater changes in whole-body metabolism, MAP, cardiac output, circulating catecholamines, and glucoregulatory hormones occur, making an atrial pacing–whole-body exercise comparison interesting, but probably not completely adequate to estimate the role of the heart in glucose or lactate disposal during moderate- to hard-intensity exercise.

**Figure 4:**
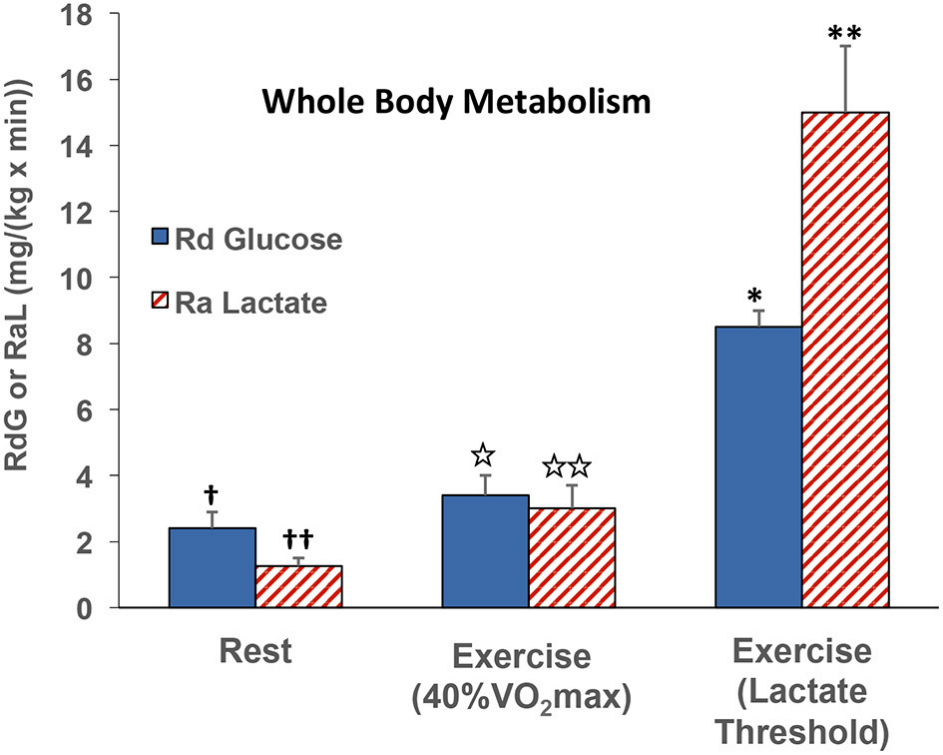
Illustration of the relationships between whole-body glucose rate of disappearance (RdG) and lactate appearance (RAL) during rest, exercise at 40% VO_2_max (47), and lactate threshold (55, 91).

At the whole-body level, comparisons of blood glucose and lactate fluxes on a mass basis (mg/kg/min) indicate that lactate rate of appearance (Ra) ~40% of that of glucose disappearance (Rd) in resting subjects (47). However, as exercise power output increases, glucose Rd increases, but lactate Ra increases relatively more, such that the two flux rates are about equal at an exercise power output that elicits about 40% VO_2_max (29, 47). At higher exercise power outputs, such as those approaching the lactate threshold (LT), the gain in whole-body lactate Ra outstrips that in glucose Rd in by almost 2-fold (Figure 4) (55, 91). Lactate Ra in excess of glucose Rd reflects the additional contribution of glycogen to glycolytic flux.

**Figure 5:**
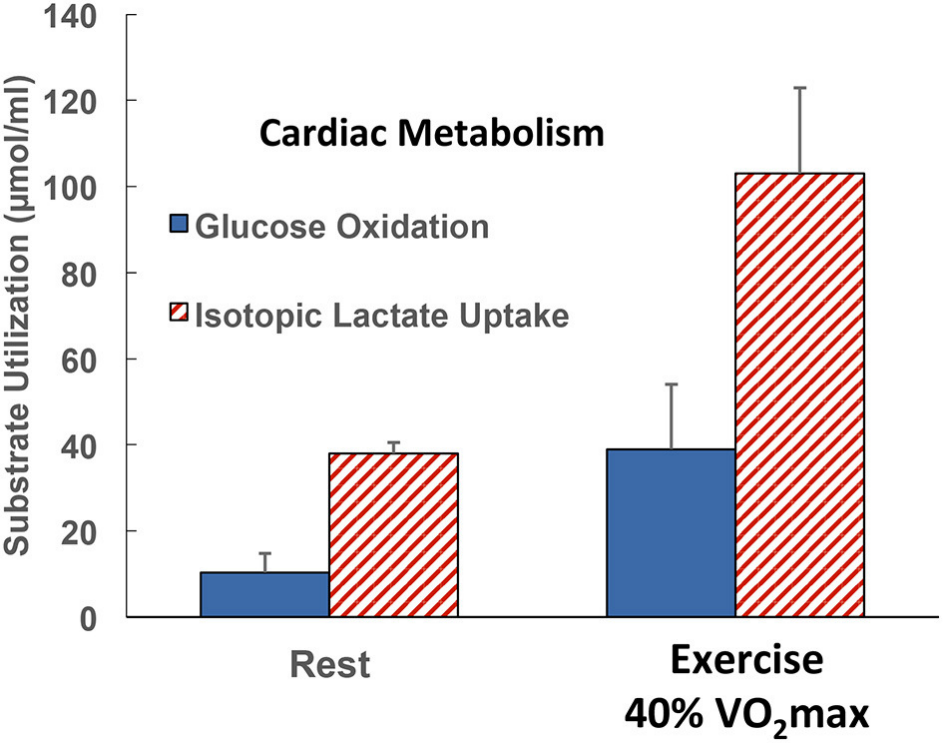
Illustration of the contributions of glucose and lactate to cardiac metabolism in healthy men studied at rest and during supine leg cycle ergometry at 40% VO_2_max (see Figure 1) (29). Results are to be compared with those in the same subjects resting and exercising at 40% of VO_2_max (see Figure 4). Results are also to be compared with those on one subject, the author, but in Figure 2 the role of fatty acids in supplying energy for cardiac metabolism is depicted. Results show that the contribution of lactate to cardiac energy need depends on arterial lactate concentration and is significantly greater than that of glucose . Results in Figure 2 also show crossover from fatty acid to lactate dependence in heart during exercise. Data from (28).

Reiterating from above, when isotope tracers of metabolites are administered, it is possible to determine whole-body metabolic flux rates. Also, if blood flow and arterial and venous drainage blood from discrete organs can be sampled, such as the jugular bulb for studies of cerebral metabolism (35), and the coronary sinus for cardiac metabolism (11, 12, 28, 29), then metabolism in those organs can be determined. For the present, we are fortunate to have data allowing simultaneous comparisons of whole-body and cardiac glucose as well as lactate flux rates in human subjects at rest and subsequently during mild to moderate-intensity (40% VO_2_max) exercise. Whole-body glucose lactate comparisons are shown in Figure 4 whereas simultaneously determined cardiac glucose–lactate comparisons are shown in Figure 5. We know of no whole-body vs. cardiac metabolism comparisons at greater exercise power outputs as assessed by either % VO_2_max or LT. Still, from the data at hand, it is apparent that the contribution of lactate to cardiac energy need is similar to or greater than that of glucose (Figure 5). Results portrayed in Figure 2 are from one subject in the same set of studies (29, 47), except that in Figure 2 the role of fatty acids is given showing crossover from cardiac fatty acid to lactate dependence in exercise.

In this and probably every other piece on cardiac metabolism, it is either understood or explicitly stated that the hard and enduring work of the heart is a most impressive feature of mammalian physiology (72). With data at hand as portrayed in Figure 5 and the original source (29), results were that lactate uptake was disposed of as oxidation whereas glucose oxidation approximated 50% of uptake in resting subjects and 70% during exercise. For resting subjects, the difference between cardiac glucose uptake and oxidation could be attributed to glycogen synthesis. However, the glucose uptake–oxidation difference during exercise is harder to explain. Could the difference be attributed to the presence of cardiac glycogen turnover and net utilization during exercise as seen in studies on laboratory rodents (92)? Or, could another amazing feature of cardiac metabolism have been operant; is it possible that cardiac glycogen storage occurs during physical exercise? Regrettably, there were no means to measure cardiac glycogen content or turnover under the conditions studied, so veracity of the alternative explanations could not be assessed.

## Summary

Lactate shuttling between producer (driver) and consumer (recipient) cells requires the presence of cell–cell and intracellular lactate shuttles that fulfill at least three purposes: lactate is (1) a major energy source, (2) the major gluconeogenic precursor, and (3) a signaling molecule. Lactate production occurs during rest and exercise under fully aerobic conditions (26, 28, 60, 93) and increases exponentially as exercise power output increases (55). There is no evidence that oxygen inadequacy gives rise to lactate production and accumulation in resting or exercising subjects, even in the hypoxia of high altitude (54). Rather, there is abundant evidence that lactate production occurs in fully aerobic tissues and organs (26, 28, 35, 51, 60, 93). Importantly, results of studies on cardiac metabolism in human subjects using coronary sinus catheterization and isotope tracer studies showed simultaneous cardiac lactate consumption, production, and oxidation at rest and during cardiac work induced by atrial pacing or physical exercise. Simultaneous measurements of working muscle, whole-body, and cardiac metabolism show lactate shuttling between working muscle and heart (12, 26, 27, 29, 46, 47). As predicted from studies on skeletal muscle (8), [(a-CS) Metabolite (Blood Flow)] measurements show crossover from lipid (fatty acid) to carbohydrate (lactate)–based metabolism with increments in cardiac work (Figure 2). The contribution of cardiac to whole-body metabolism appears to scale to whole-body metabolic rate and represents 5% at rest to 15% during atrial pacing (12) or easy (40% VO_2_max) exercise (29, 47). The contribution of cardiac to whole-body metabolism during hard exercise has yet to be determined, but is likely not much greater than during easy exercise because increasing muscle mass, recruitment of type 2 glycolytic fibers, and catecholamine stimulation during hard and intense exercise (55) likely overwhelm the heart's ability to clear lactate from the circulation. Lactate shuttling from muscle to heart fuels it during rest or exercise when lactate is the dominant energy source (Figures 2, 5). Although limited by small relative mass, the heart plays a significant role during rest and exercise.

Original articulation of the lactate shuttle (6, 15) was based on the contemporaneous observations of muscle cell heterogeneity and posited the exchange of lactate between driver and recipient cells, organs, and tissues. In view of data on cardiac blood flow during the cardiac cycle, it is now possible to posit presence of a cardiac or intra-cardiac lactate shuttle in which interruption of blood flow and rapid glycolysis occur during the isotonic phase of systole with the return of flow and oxidative metabolism during diastole. Whether the hypothesis of a phasic cardiac lactate shuttle is viable remains to be determined. Regardless, it is clear that lactate released from working muscles (47) or released from the integument (94) as a consequence of sympathetic nervous system activation and catecholamine-stimulated glycogenolysis (1) can fuel the heart (Figure 2). And lastly, as in the instances of whole-body exercise (8) or working muscle (27), increases in cardiac work cause a crossover (shift) from lipid- to carbohydrate-derived fuel energy sources (Figures 2, 5).

## Author Contributions

The paper was conceived and written by GB.

## Conflict of Interest

The author declares that the research was conducted in the absence of any commercial or financial relationships that could be construed as a potential conflict of interest.
